# Awake prone positioning in nonintubated spontaneous breathing ICU patients with acute hypoxemic respiratory failure (PRONELIFE)—protocol for a randomized clinical trial

**DOI:** 10.1186/s13063-021-05991-2

**Published:** 2022-01-10

**Authors:** L. Morales-Quinteros, M. J. Schultz, A. Serpa-Neto, M. Antonelli, D. L. Grieco, O. Roca, N. P. Juffermans, C. de Haro, D. de Mendoza, Ll. Blanch, M. Camprubí-Rimblas, Gemma Gomà, A. Artigas-Raventós, for the PRONELIFE collaborative group

**Affiliations:** 1 Department of Intensive Care Medicine, Hospital Universitari Sant Pau, Barcelona, Spain; 2 Translational Research Laboratory, Institut d’Investigació i Innovació Parc Taulí I3PT Universitat Autònoma de Barcelona Sabadell, Parc del Tauli- 08208 Sabadell, Barcelona, Spain; 3 Department of Intensive & Laboratory of Experimental Intensive Care and Anesthesiology, Amsterdam UMC, location “AMC”, Amsterdam, The Netherlands; 4 Mahidol Oxford Tropical Medicine Research Unit (MORU), Mahidol University, Bangkok, Thailand; 5 Nuffield Department of Medicine, University of Oxford, Oxford, UK; 6 Department of Critical Care Medicine, Hospital Israelita Albert Einstein, Sao Paulo, Brazil; 7 Department of Intensive Care Medicine, Austin Hospital and University of Melbourne, Melbourne, VIC Australia; 8 Department of Anesthesiology and Intensive Care Medicine, Catholic University of the Sacred Heart, “A. Gemelli” University Hospital, Rome, Italy; 9 Department of Intensive Care Medicine & Vall d’Hebron Research Institute, Vall d’Hebron University Hospital, Universitat Autònoma de Barcelona, Barcelona, Spain; 10 Centro de Investigación Biomédica en Red en Enfermedades Respiratorias, Instituto de Salud Carlos III, Madrid, Spain; 11 Department of Intensive Care Medicine, Onze Lieve Vrouwe Gasthuis (OLVG) Hospital, Amsterdam, The Netherlands; 12 Department of Intensive Care Medicine, Corporación Sanitaria Universitaria Parc Tauli, Barcelona, Spain; 13 Department of Intensive Care Medicine, Sagrat Cor University Hospital, Grupo Quironsalud, Barcelona, Spain

**Keywords:** ICU, Acute respiratory failure, Hypoxemia, Prone position, Awake prone positioning, Invasive ventilation, Intubation, Invasive ventilation, Randomized controlled trial

## Abstract

**Background:**

It is uncertain whether awake prone positioning can prevent intubation for invasive ventilation in spontaneous breathing critically ill patients with acute hypoxemic respiratory failure. Awake prone positioning could benefit these patients for various reasons, including a reduction in direct harm to lung tissue, and prevention of tracheal intubation-related complications.

**Design and methods:**

The PRONELIFE study is an investigator-initiated, international, multicenter, randomized clinical trial in patients who may need invasive ventilation because of acute hypoxemic respiratory failure. Consecutive patients admitted to participating ICUs are randomly assigned to standard care with awake prone positioning, versus standard care without awake prone positioning. The primary endpoint is a composite of tracheal intubation and all-cause mortality in the first 14 days after enrolment. Secondary endpoints include time to tracheal intubation and effects of awake prone positioning on oxygenation parameters, dyspnea sensation, and complications. Other endpoints are the number of days free from ventilation and alive at 28 days, total duration of use of noninvasive respiratory support, total duration of invasive ventilation, length of stay in ICU and hospital, and mortality in ICU and hospital, and at 28, 60, and 90 days. We will also collect data regarding the tolerance of prone positioning.

**Discussion:**

The PRONELIFE study is among the first randomized clinical trials investigating the effect of awake prone positioning on intubation rate in ICU patients with acute hypoxemic failure from any cause. The PRONELIFE study is sufficiently sized to determine the effect of awake prone positioning on intubation for invasive ventilation—patients are eligible in case of acute hypoxemic respiratory failure without restrictions regarding etiology. The PRONELIFE study is a pragmatic trial in which blinding is impossible—however, as around 35 ICUs worldwide will participate in this study, its findings will be highly generalizable. The findings of the PRONELIFE study have the potential to change clinical management of patients who may need invasive ventilation because of acute hypoxemic respiratory failure.

**Trial registration:**

ISRCTN ISRCTN11536318. Registered on 17 September 2021. The PRONELIFE study is registered at clinicaltrials.gov with reference number NCT04142736 (October, 2019).

## Background

Acute hypoxic respiratory failure represents one of the most common reasons for intensive care unit (ICU) admission [1]. Initial management of hypoxemic patients should involve immediate administration of simple supplemental oxygen via a nasal prong or a non-rebreather mask, or more complex forms of respiratory support like high-flow nasal oxygen (HFNO) oxygen or noninvasive ventilation (NIV), depending on severity of hypoxic respiratory failure and the underlying cause, but also patient characteristics and the availability of oxygen interfaces. Unfortunately, patients with acute hypoxemic respiratory failure often need to proceed with invasive ventilation. While life-saving, this intervention comes with disadvantages, including ventilator-induced lung injury and respiratory muscle waist, but also intubation-related side-effects. In addition, intubated patients often need sedation, which has side-effects of its own.

In intubated invasively ventilated ICU patients, prone positioning can be used to improve oxygenation, and this intervention has been shown to improve outcome in patients with severe ARDS. This survival benefit could be mediated, at least in part through a reduction in direct harm to lung tissue, as regional differences in lung aeration, compliance, and shear strain are minimized by this intervention [2, 3]. In theory, nonintubated patients could also benefit from prone positioning [4, 5], a strategy named “awake prone positioning” [6, 7]. Some evidence for benefit of awake prone positioning comes from a handful of studies, mostly case reports and single-center observation case series [5, 8, 9]. The findings thus far show that awake prone positioning can indeed improve oxygenation and also reduce dyspnea sensation. Besides that, awake prone positioning seems to be well-accepted and easy to perform, and to have relatively few side-effects [10].

In the absence of sufficient randomized clinical trial evidence, we designed the PRONELIFE study, a pragmatic study that compares standard care with awake prone positioning versus standard care without awake prone positioning in patients with acute hypoxemic respiratory failure from any cause. We hypothesize that awake prone positioning reduces the need for intubation.

## Methods

### Design

The “PRone positioN in patients with spontanEous ventiLation and acute hypoxemic respIratory FailurE” (PRONELIFE) study is a pragmatic, investigator-initiated, international and multicenter, two-arm, superiority randomized clinical trial in patients with acute hypoxemic respiratory failure from any cause. The study will be conducted according to the Declaration of Helsinki principles as stated in the current version of Fortaleza, Brazil, 2013 [11]. The Institutional Review Board of the Sagrat Cor University Hospital, Barcelona, Spain, approved the study protocol (reference number 2019/68-UCI-HUSC). The study is registered at www.clinicaltrials.gov (NCT04142736, October 2019). The PRONELIFE study is planned to be performed in the 35 ICUs worldwide. Patients will be provisionally included under a strategy of deferred consent, for reasons as explained below.

### Consort diagram

**Fig. 1:**
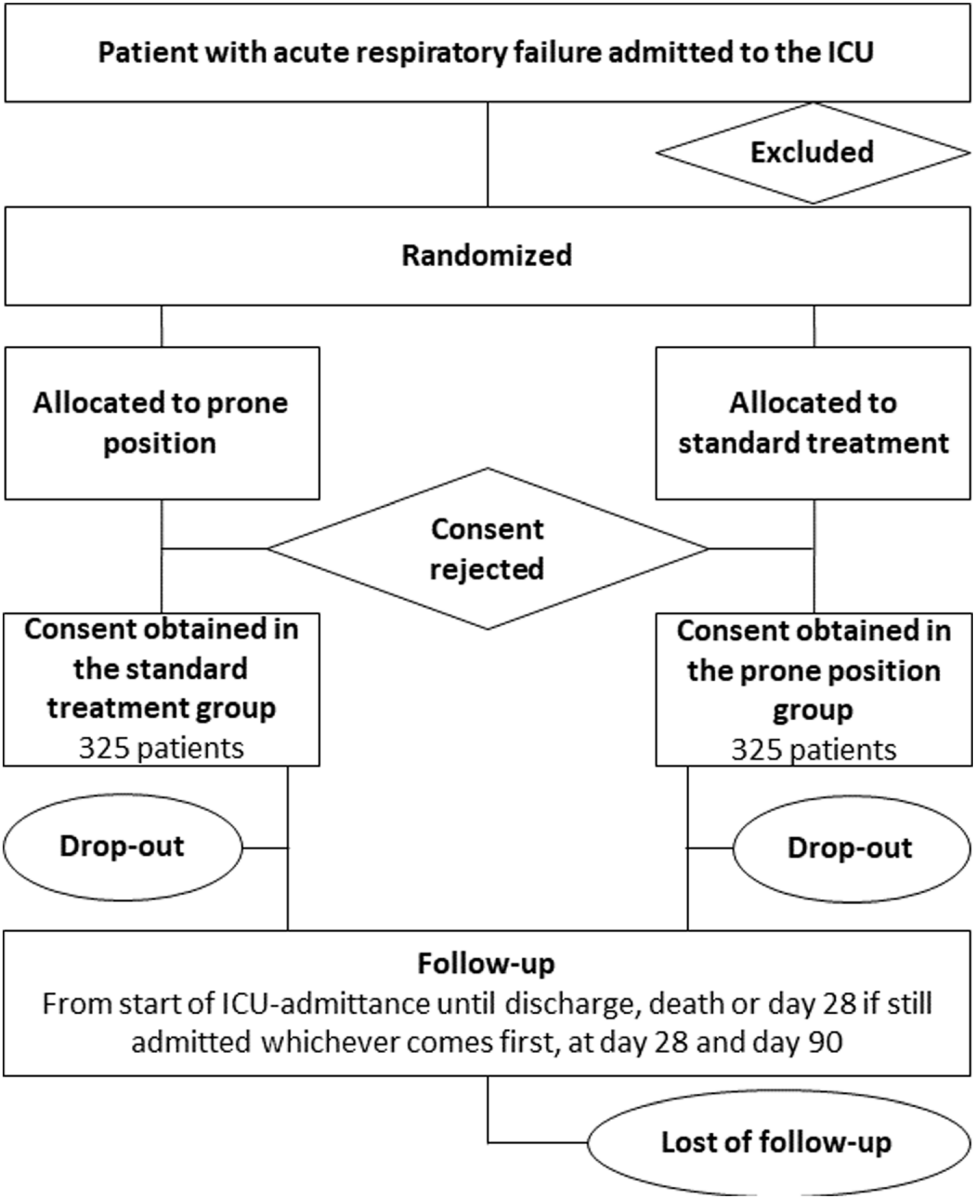
Consolidated Standards of Reporting Trials (CONSORT) diagram

**Fig. 2:**
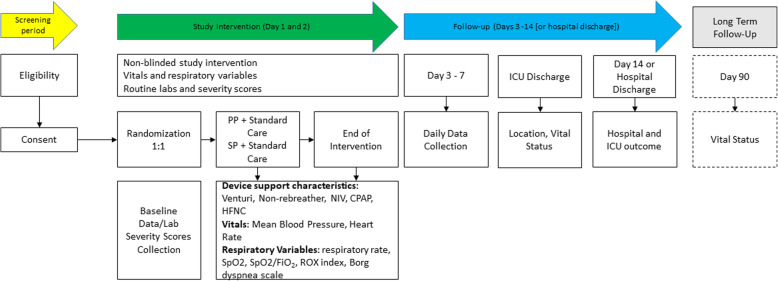
Study overview

The Consolidated Standards of Reporting Trials (CONSORT) [12] diagram of the PRONELIFE study is presented in Fig. 1. Consecutive patients admitted to the participating ICUs are screened for eligibility. Demographic data are registered regardless of meeting enrollment criteria. If excluded from participation, the reason(s) for exclusion will be reported. The study overview is presented in Fig. 2.

### Inclusion and exclusion criteria

Patients admitted to the ICU with acute hypoxemic respiratory failure from any cause will be eligible for participation, unless prompt intubation is irrepressible (Table 1). Acute respiratory failure is defined as the need for supplementary oxygen with an FiO_2_ of at least 40% by Venturi facemask, HFNO, NIV, or CPAP needed to achieve and maintain an SpO_2_ of between 88 and 92%.

**Table 1:** Contraindication for awake prone position

• Suspected increased intracranial pressure (e.g., severe brain injury) • Hemoptysis • Vomiting • Recent abdominal wound (less than 15 days) • Tracheal surgery or sternotomy during the previous 15 days • Facial trauma or facial surgery during the previous 15 days • Deep venous thrombosis treated for less than 2 days • Cardiac pacemaker inserted in the last 2 days • Unstable spine, femur, or pelvic fractures • Hemodynamic instability (defined by a systolic blood pressure below 90 mmHg, mean blood pressure below 65 mmHg, or requirement for vasopressor) • Pregnant women • Presence of chest tube	

The PRONELIFE study has the following exclusion criteria: age < 18 years, having any contraindication for prone positioning (Table 2), previous participation in this trial, and participation in other interventional trials with the same primary endpoint. We also exclude pregnant patients, patients who refuse intubation, and those only receiving comfort care. As patients will be provisionally included under a strategy of deferred consent, there is no need for written informed consent at start of the study—however, if consent is not obtained within 48 h, a patient drops out of the study and will be replaced.

**Table 2:** Borg dyspnea scale

0	Nothing at all
0.5	Very, very slight (just noticeable)
1	Very slight
2	Slight breathlessness
3	Moderate
4	Somewhat severe
5	Severe breathlessness
6	
7	Very severe breathlessness
8	
9	Very, very severe (almost maximal)
10	Maximal

### Study endpoints

The primary endpoint is the composite of tracheal intubation and all-cause mortality within 14 days after enrollment.

Secondary endpoints are the effects of awake proning on the following clinical outcome variables:

- Time to tracheal intubation (only in patients who need invasive ventilation);
- Oxygenation parameters;
- Dyspnea sensation (while awake in prone versus supine position);
- The number of days free from invasive ventilation and alive at day 28;
- Duration of use of noninvasive ventilatory support, including the use of a non-rebreather mask, CPAP, high-flow nasal oxygen (HFNO) oxygen, or noninvasive ventilation (NIV);
- Duration of invasive ventilation;
- Ventilation-free days (VFD) at 28 days from ICU admission, defined as the number of days alive and free from IMV during the first 28 days from start of IMV;
- Length of stay in ICU and hospital;
- Mortality in ICU and hospital, and at 28, 60, and 90 days; and
- Tolerance of prone positioning (only in patients in the prone positioning group).

### Randomization and blinding

Randomization will be performed using a dedicated password-protected website and will be balanced per center. Central randomization with the use of a permutated-block randomization list (with block sizes of 4 to 8) will be used. Participants will be allocated to the prone positioning or standard care on a 1:1 ratio. By the nature of the intervention, it will not be possible to blind clinicians to whether a participant has been randomized to awake proning or standard care. Interventions will be blinded to data analysts and outcome assessors.

### Prone positioning

The study intervention will last for at least 48 h and is divided in 4 blocks of 6 h each: patients will be placed in the prone position for up to 2 h, which can be prolonged if the patient feels comfortable, but could also be interrupted if a patient meets any of the discontinuation criteria which are any of the following:

- Developing a contraindication (Table 1);
- Worsening of dyspnea (at any time, according to predefined criteria, as described in Table 2);
- A further and sustained drop in SpO_2_ refractory to an increase in FiO_2_; nausea or vomiting; and
- Increasing hemodynamic instability that is unrelated to sedatives (if given) and cannot be corrected by vasopressor or inotrope infusion (as described in Table 3)

**Table 3:** Hemodynamic instability definition

Defined as any of the following not responding to fluid resuscitation:	
• Systolic arterial pressure < 90 mmHg, or • Mean arterial pressure < 65 mmHg, or • Increased needs of vasopressor agents, or • ECG evidence of ischemia or significant uncontrolled ventricular arrhythmia	

During the change in position, from supine to prone or from prone to supine, FiO_2_ will be increased by 25% above baseline. Each change in position is guided by two healthcare workers and an attending physician, but more healthcare workers could be needed. While the patient remains in a prone position, skin protection will be used to avoid pressure sores. Also, the application of cushions will enhance patient tolerance. Arms can be at the side, in a swimmer’s position, and can be moved to increase comfort.

Food and comfort breaks are planned while patients are in supine. If the patient is receiving enteral or oral feeding, this is interrupted from 1 h before prone until a patient is in a supine position.

The best-fitting and most-tolerated oxygen interface will be used in the prone position—this could be different from patient to patient, and different from what is used in the supine position, and could differ between patients but also institutions (i.e., depending on the availability of masks with or without a reservoir bag and with or without the Venturi system, HFNO, CPAP or NIV).

### Standard of care

In all patients, whether receiving prone positioning or not, the best standard of care is provided, according to the standard care by the local teams.

When in a supine position, the patient will be placed in 30–45° semi-recumbent position but this can be changed for the comfort of the patient to supine, semi-sitting, sitting, or a lateral decubitus position.

Vitals parameters, including SpO_2_ and the SpO_2_/FiO_2_, are continuously monitored. The oxygenation target ranges for SpO_2_ are 88 to 92%; this is 7 to 8 kPa for PaO_2_. For patients in whom the risk of potentially dangerous hypoxemia could become unacceptable during the study (e.g., in patients who develop cardiac ischemia due to cardiac infarction or failed revascularization, or severe untreatable anemia such as with Jehovah’s Witnesses), oxygenation target ranges can be higher, 94 to 96% for SpO_2_ and 9 to 11.5 kPa for PaO_2_ [13–15].

Opiates and benzodiazepines are allowed at low dosages to improve comfort.

### Intubation criteria

The decision to continue with invasive mechanical ventilation is based on clinical judgment rather than isolated gasometrical criteria. Any of the following criteria should be considered for proceeding to endotracheal intubation:

- Respiratory or cardiac arrest;
- Respiratory pauses;
- Altered level of consciousness such as uncontrolled agitation not responding to medical treatment, or a drop in the Glasgow Coma Score;
- Evidence of exhaustion such as an unacceptable increase in use of accessory muscles or thoracoabdominal paradox;
- Inability to clear secretions from the airway in patients with abundant sputum production, or evidence of aspiration; or
- Hemodynamic instability as defined in Table 3

In addition, the presence of 2 of the following criteria within 1 h of start noninvasive ventilatory support:

- Respiratory rate > 35 breaths/min, or increased respiratory rate from the baseline;
- Not improving or increased dyspnea;
- pH < 7.30 or less from its baseline, or PaCO_2_ > 20% from the baseline value; or
- SpO_2_ < 88%

### Weaning from the noninvasive oxygen delivery system

Weaning from the oxygen therapy delivery system will be done according to local protocols and preferences. Table 4 provides guidance for oxygen requirements coupled with the reduction in gas flow rates of the HFNO.

**Table 4:** High-flow nasal cannula scheme for oxygen requirements coupled with gas flow rates

FiO_2_	21–30%	30–40%	40–60%	60–100%
Flow	30 L/min	30–40 L/min	40–50 L/min	50–70 L/min

In case the patient is using NIV, sessions could be interrupted when the respiratory rate is < 25 breaths/min and the FiO_2_ < 40% (for a SpO_2_ 88%)

### Invasive ventilation

If patient continues with invasive ventilation, settings are chosen in line with the local guidelines for invasive ventilation. The use of lung-protective ventilation with a low tidal volume and low pressures is advocated. Also, sufficient levels of PEEP should be used, and prone positioning is to be applied if a patient develops severe hypoxemia, defined as PaO_2_/FiO_2_ < 150 mmHg at a minimum FiO_2_ of 60% and 5 cmH_2_O.

### Fluid regimens

We advise to use a restricted fluid strategy, i.e., targeting a neutral cumulative fluid balance as soon as a patient can be weaned of vasopressors. Crystalloid infusions are preferred over colloid infusions.

### Sedation

In patients under invasive ventilation, the local guideline for sedation is to be followed, and preferably consists of combinations of use of analgo-sedation over hypno-sedation, use of bolus over continuous infusion of sedating agents, and the use of sedation scores (e.g., 3 times per day, and using a Richmond Agitation Sedation Scale) [16, 17]. Also, the level of pain is to be determined, e.g., by using the Numeric Rating Scale, the Visual Analog Scale (VAS), the Critical Care Pain Observation Tool (CCPOT) or Behavioral Pain Scale (BPS).

### Weaning from invasive ventilation

Weaning from invasive ventilation follows local protocols and preferences. In all patients, it should be tested whether the patient accepts assist ventilation at least two times a day; this should also be tried when the patient shows respiratory muscle activity during assist ventilation. The attending physician decides when to extubate a patient, based on general extubation criteria (i.e., responsive and cooperative, adequate cough reflex, adequate oxygenation with FiO_2_ ≤ 0.4, hemodynamically stable, no uncontrolled arrhythmia and a rectal temperature > 36 °C, and after successfully passing a spontaneous breathing trial (SBT) with a T-piece or ventilation with minimal support (pressure support level < 10 cmH_2_O) and FiO_2_ ≤ 0.4). In case SBTs are used, an SBT is judged as successful when the following criteria are met for at least 30 min, and the attending physician takes the final decision for extubation:

- Respiratory rate < 35 breath/min;
- Peripheral oxygen saturation > 90%;
- Increase < 20% of heart rate and blood pressure; and
- No signs of anxiety and diaphoresis.

### Data collection

Data will be entered via an electronic case report from (eCRF). On ICU admission and within 48 h, demographic and baseline data and data on disease severity will be collected. Data collection includes gender, age, height, weight, date of hospital admission, date of ICU admission, cause of respiratory failure, chest X-ray, the Sequential Organ Failure Assessment (SOFA) score, the Acute Physiology and Chronic Health Evaluation II (APACHE II) score, and the Simplified Acute Physiology Score II (SAPS II).

**Fig. 3:**
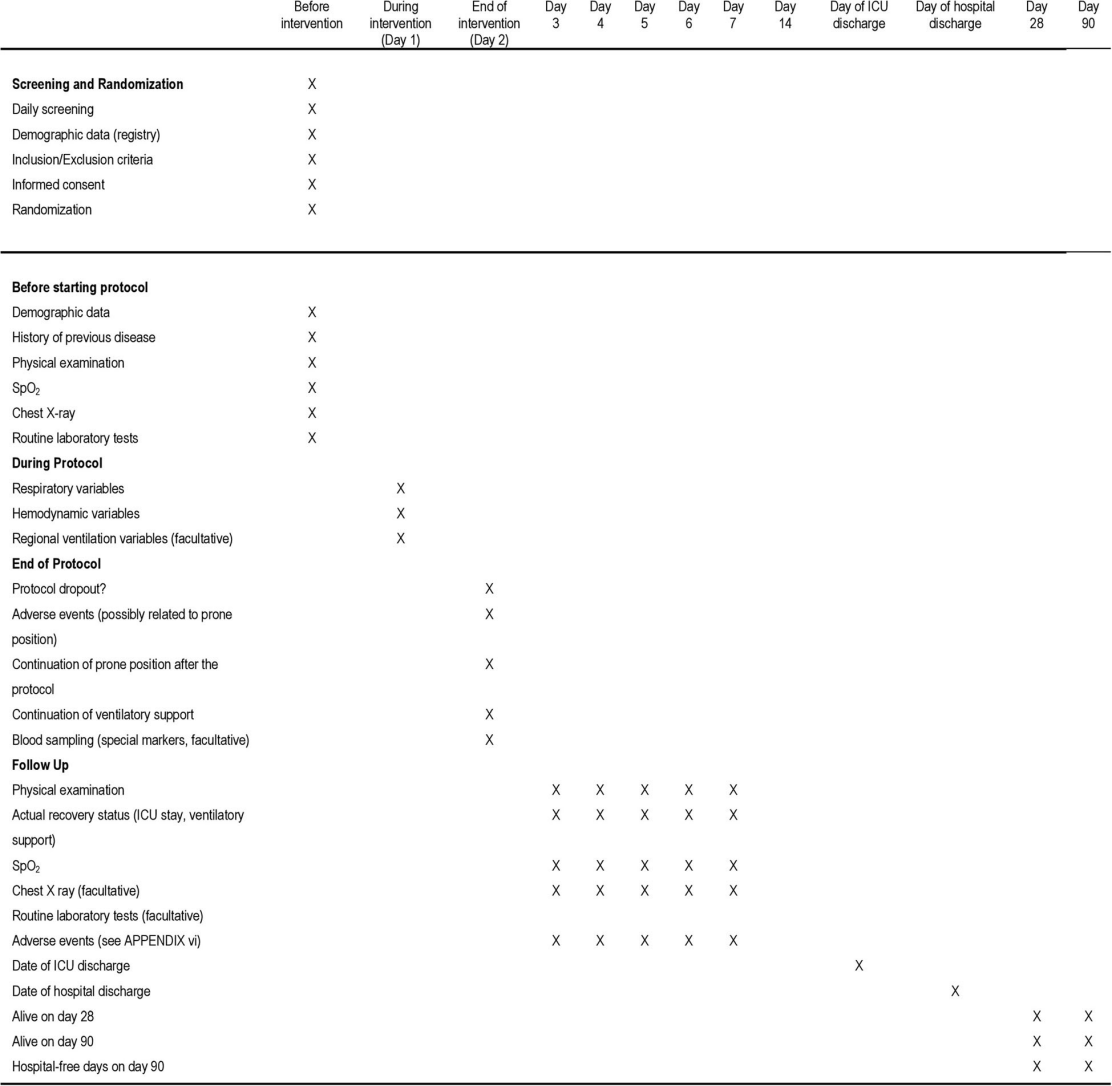
Schedule of events

Data on the standard of care (described below) will be collected daily for the first 48 h and every 6 h, up to day 28. The total number of hours spent in prone position in a day (cumulative), the number of prone sessions, and their duration is recorded. After the protocol, every day until day 14, clinical outcome variables are discharge of the ICU or death, whichever comes first. Data on duration of noninvasive respiratory support, length of stay in ICU and hospital, location of the patient (in ICU, hospital, another facility, or home), and life status (alive or deceased) will be assessed on 28 and 90 days (Fig. 3).

One hour before each position change (supine to prone and prone to supine), the following variables will be collected for the duration of the protocol: respiratory variables (respiratory rate SpO_2_, SpO_2_/FiO_2_, ROX index; SpO_2_/FiO_2_: RR, dyspnea defined according to the Borg dyspnea scale); noninvasive mean arterial pressure; heart rate; noninvasive support device parameters: Venturi facemask, HFNO, CPAP, NIV; adverse effects possibly related to prone position (hypotension, bradycardia hypoxemia, pressure ulcers, vomiting, displacements of the respiratory support device, peripheral or venous central line, orogastric or nasogastric tube, or urinary catheter), if any are recorded in the eCRF.

In case prone positioning is not tolerated, all reasons for a premature change in body position are reported.

The following parameters of the respiratory support devices will be collected within 1 h before and 1 h after randomization, and every day at a fixed time point until cessation of the respiratory support: Venturi facemask: flow (liters/min), FiO_2_; HFNO: flow (liters/min), FiO_2_; CPAP: PEEP, flow (liters/min), tidal volume, FiO_2_; NIV: PEEP, pressure support over PEEP, flow (liters/min), tidal volume, FiO_2_.

If the patient meets the criteria for intubation, the reasons for intubation are documented in the eCRF.

Data on the administration on the administration of steroids, Remdesivir, Tocilizumab, Heparin/low molecular weight heparin, and antihypertensive medications are recorded. Fluid balance is collected from the nursing chart.

### Power calculation

We will include a total of 650 patients. The required sample size is calculated using data from two multicenter randomized controlled trials reporting intubation rates in patients with acute hypoxemic respiratory failure [18, 19]. It was conservatively assumed that the rate of tracheal intubation will be 45% in the standard treatment group. Accordingly, 650 patients (*n* = 325 per group) would provide 80% power to detect a relative risk of 0.75 for the primary endpoint at a 2-sided *α* level of 0.05, assuming a dropout rate of 10%. After enrollment of 70% of the patients, an unblinded sample size reestimation will be performed, and sample size could be increased to a maximum of 1000 patients if needed.

### Deferred informed consent

For this study, we will include patients using a strategy using deferred informed consent because we explicitly want to randomize and start ventilation according to randomization within 1 h after ICU admission. Nevertheless, the legal representative’s written informed consent will be requested as soon as possible thereafter and never later than 48 h after randomization. If informed consent is not obtained within those 48 h, or if a legal representative denies participation within this time frame, the patient is excluded, and data will no longer be used, nor will this patient be counted for the sample size of 325 inclusions in each group (that is the provisionally included patient for whom informed consent is not obtained within the time frame of 48 h is “replaced” by a new patient until the total number of 325 patients in each arm is definitively included). On the consent form, participants will be asked if they agree to use their data should they choose to withdraw from the trial. Participants will also be asked for permission for the research team to share relevant data with people from the Hospitals taking part in the research or from regulatory authorities. This trial does involve collecting biological specimens.

### Statistical analysis

Statistical analysis will be based on the intention-to-treat principle. We will also perform a per-protocol analysis, comparing patients who received awake prone positioning and patients who received standard of care.

Continuous normally distributed variables will be expressed by their mean and standard deviation or when not normally distributed as medians and their interquartile ranges. Categorical variables will be expressed as *n* (%). Where appropriate, statistical uncertainty will be expressed by the 95% confidence levels.

The primary outcome, the number of days free of ventilation at day 14, will be analyzed using Cox’s regression. The possible imbalance between groups will be modeled in the Cox model. To further compare groups, Student’s *t* test will be used. If continuous data is not normally distributed, the Mann-Whitney *U* test will be used. Categorical variables will be compared with the chi-square test or Fisher’s exact tests. Time-dependent data will be analyzed using a proportional hazard model adjusted for possible imbalances of patients’ baseline characteristics. No interim analysis will be conducted.

The level and pattern of missing data in the baseline variables and outcomes will be established by forming appropriate tables and the likely causes of any missing data will be investigated. If necessary, multiple imputation or Bayesian methods for missing data will be used.

A *P* value < 0.05 is considered statistical significance. The analysis will be performed with R statistics version 3.0.2 (R Foundation, Vienna, Austria).

### Study organization

The steering committee comprises the principal investigator, the coordinating investigators, and eight international experts in ventilatory support in critically ill patients.

The coordinating investigator is responsible for administrative management and communication with the local investigators and assists at the participating clinical sites in trial management, record keeping, and data management. The coordinating investigators help set up local training in the participating ICUs to ensure the study is conducted according to the ICH-GCP guidelines, guarantee data collection integrity, and ensure timely completion of the case report forms. The local investigators provide structural and scientific leadership. They guarantee the integrity of data collection and ensure timely completion of the case report forms.

An independent monitor will be installed. Remote monitoring utilizing queries on the database will be done by a statistician and analyzed by the monitor to signalize early aberrant patterns, trends, issues with consistency or credibility, and other anomalies. On-site monitoring will comprise controlling the presence and completeness of the research dossier and the informed consent forms, and source data checks will be performed in the files of 25% of the patients.

An independent Data and Safety Monitoring Board (DSMB) watches over the ethics of conducting the study under the Declaration of Helsinki and monitors safety parameters and the overall conduct of the research. The DSMB is composed of three independent individuals (Prof. Arthur Slutsky, Prof. Claude Guerin, and Dr. Tài Pham). The DSMB will meet by conference calls. The first meeting is scheduled after the first 100 patients. After this meeting, the DSMB will meet every 6 months.

All unexpected adverse events will be reported to the DSMB. Any report or advice of the DSMB will be sent to the sponsor of the study, the Institut d'Investigació i Innovació Parc Taulí (I3PT), Sabadell, Spain. Should the sponsor decide not to implement the advice of the DSMB fully, the sponsor will send the recommendation to the reviewing Institutional Review Board, including a note to substantiate why (part of) the advice of the DSMB will not be followed. All substantial amendments will be notified to the Sponsor Ethical Review Board first and the Funder. Then the PI will notify the centers, and a copy of a revised protocol will be sent back to the PI to add to the Investigator Site File. Any deviations in the protocol will be fully documented using a breach report form. Non-substantial amendments (typing errors and administrative changes) will not be notified to the Sponsor Ethical Review Board. The protocol will be updated in the clinical trial registry.

## Discussion

PRONELIFE is among the first clinical trials of awake prone positioning in patients with acute hypoxemic respiratory failure that will recruit a sufficient number of patients to test the hypothesis that this intervention prevents tracheal intubation. PRONELIFE will also collect data regarding other patient-centered outcomes, including duration of ventilation if needed, length of stay in ICU and hospital, and mortality. We will also study the tolerability of awake prone positioning and its effects on oxygenation.

Invasive ventilation is a life-support strategy associated with serious complications that may result in significant morbidity and mortality. In addition, endotracheal intubation is a high-risk procedure in critically ill patients, with major complications like severe hypoxemia, cardiovascular instability, and even cardiac arrest [20–22]. The primary endpoint of this study is a composite of the need for invasive ventilation and death within the first 14 days. We decide to use this composite endpoint, hypothesizing that the intervention under study will impact two outcomes that are invariable related. Using a composite outcome will also increase the event rate, improve the statistical efficiency for sample size calculation, and improve the study’s precision. We chose the need for invasive ventilation and death within the first 14 days because we hypothesize that the impact of awake prone positioning will not last beyond the first 2 weeks. Of note, we will report the two components of the composite as two secondary endpoints.

Evidence for the benefit of awake prone positioning in nonintubated ICU patients is scarce. Its first use was described in a case series of patients with hypoxemic respiratory failure, showing improvements in oxygenation [8]. Improvements in oxygenation were also reported in two studies in patients with severe hypoxemia after lung transplantation [5, 23]. A rapidly growing number of mainly small observational studies describe the use of awake prone positioning in patients with spontaneous breathing with acute respiratory failure in whom hypoxemia is refractory to supplementary oxygen. It has been shown that awake prone positioning can improve oxygenation within minutes [24–28], and the effects are maintained for up to 1 h after turning back to supine and disappear mostly after 6 to 12 h [29]. A total of 15 studies, representing 449 patients, were recently used in one meta-analysis [30], assessing the change in oxygenation (i.e., PaO_2_/FiO_2_ ratio, PaO_2_, and SpO_2_) induced by awake prone positioning. Two recent studies failed to show benefit of awake prone positioning in patients with acute respiratory failure [31, 32]. Of note, one of these studies had an observational design, and it remained uncertain whether the intervention was used as a routine or life-saving therapy [32]. The other study was a randomized clinical trial that was underpowered for sample size estimation [31]. In both studies, patients were only included patients with COVID-19 infection. Evidence suggests that direct versus indirect causes of lung injury possess different pathophysiologic characteristics that might impact both the progression and outcome of ARDS [33–35]. Therefore, the prone position could affect mortality, and its impact may vary according to the etiology of lung injury. Also, in both studies, the intubation criteria were not clearly defined. Our study will include different etiologies of acute respiratory failure, and we have clearly described intubation criteria.

Awake prone positioning is an attractive intervention for several reasons. First, many patients with hypoxemic acute respiratory failure seem to tolerate awake prone positioning relatively well [24, 36, 37]. Awake prone positioning is also associated with few complications. Data are limited, but thus far, no severe adverse events have been described in the literature. Pressure sores, frequently developing in intubated ICU patients [38] and with higher morbidity [39], have only been rarely described in patients who received awake prone positioning [9, 31]. Intolerance to awake prone positioning could be related to musculoskeletal discomfort [24, 26, 37, 40], nausea and vomiting [26], cough [37], and anxiety [40]. Our study will focus on these aspects.

PRONELIFE will also collect other important and patient-centered endpoints such as dyspnea sensation, duration of use of noninvasive ventilatory support, and duration of invasive ventilation and the classical ICU endpoints like the length of ICU and hospital stay, and various mortality rates.

We anticipate the PRONELIFE study to be highly feasible and easy to conduct as the study procedures are straightforward, without difficult or complex interventions making the treatment of patients clear and easy. Furthermore, the PRONELIFE study uses a deferred consent strategy to include patients rapidly in the study and those admitted during evenings or nights when researchers and family members are often not around to ask for informed consent. This will create a study population representative of the average ICU population. Also, PRONELIFE will be performed in the ICUs of different hospitals worldwide, increasing the generalizability of its findings. Furthermore, we include patients with any cause of respiratory failure.

One important limitation of PRONELIFE is that blinding is not possible due to the nature of the intervention tested, which could induce bias. However, the indication for invasive ventilation, which directly influences the primary outcome, is clearly stated, and all analyses will be performed in a blinded fashion. Second, we foresee that another limitation could derive from the time under prone position a patient with spontaneous breathing could tolerate continuously. Knowing from studies that the benefit of prone position in ARDS could depend on its duration [41], the protocol design will count the total time of prone positioning for each patient. Although patients randomized to the intervention will have 2 h under prone position, this strategy could be further prolonged if the patient feels comfortable.

The results of PRONELIFE have the potential to change clinical practice in terms of how we manage patients with acute hypoxemic respiratory failure.

In conclusion, the PRONELIFE study is an international, investigator-initiated randomized clinical trial that is adequately powered to test the hypothesis that awake prone positioning benefits ICU patients with acute hypoxemic respiratory failure.

## Trial status

Pending to start recruiting.

Date recruitment will begin in 1 February 2022.

Recruitment will be completed in February 2024.

Protocol Version 5.4, 16 July 2021.

## Data Availability

Morales-Quinteros, Serpa Neto, Artigas, and Schultz had full access to all data in the study and take responsibility for the integrity of the data and the accuracy of the data analysis; the members of the Steering Committee for PRONELIFE Collaborative Group vouch for the accuracy and completeness of the data and for the fidelity of the study to the protocol. All data needed to evaluate the conclusions of the trial will be present and tabulated in the final manuscript. Individual de-identified raw data will be available from the corresponding author on reasonable request during the first year after publication of the primary manuscript arising from this study.

## Abbreviations

APACHE II: Acute physiology and chronic health evaluation II
ARDS: Acute respiratory distress syndrome
BPS: Behavioral pain scale
CCPOT: Critical care pain observation tool
CONSORT: Consolidated standards of reporting trials
CPAP: Continuous positive airway pressure
DSMB: Data and safety monitoring board
eCRF: Electronic case report form
FiO_2_: Fraction of inspired oxygen
HFNO: High-flow nasal oxygen
ICH-GCP: International conference on harmonization-good clinical practice
IMV: Invasive mechanical ventilation
ICU: Intensive care unit
NIV: Noninvasive ventilation
PaCO_2_: Partial pressure of arterial carbon dioxide
PaO_2_: Partial pressure of arterial oxygen
PEEP: Positive end-expiratory pressure
ROX: Respiratory OXygenation
SAPS II: Simplified acute physiology score II
SBT: Spontaneous breathing trial
SOFA: Sequential organ failure assessment
SpO_2_: Peripheral capillary oxygen saturation
VAS: Visual analog scale
VFD: Ventilator-free days
